# Considering γ’ and Dislocation in Constitutive Modeling of Hot Compression Behavior of Nickel-Based Powder Superalloy

**DOI:** 10.3390/ma18204680

**Published:** 2025-10-12

**Authors:** Liwei Xie, Jinhe Shi, Jiayu Liang, Dechong Li, Lei Zhao, Qian Bai, Kailun Zheng, Yaping Wang

**Affiliations:** 1 School of Mechanical Engineering, Dalian University of Technology, Dalian 116024, China; 2028433737@mail.dlut.edu.cn (L.X.); shijinhe@mail.dlut.edu.cn (J.S.); 15128205601@163.com (J.L.); ldc7196@mail.dlut.edu.cn (D.L.); leizhao@dlut.edu.cn (L.Z.); zhengkailun@dlut.edu.cn (K.Z.); 2 Department of Materials Science and Engineering, Norwegian University of Science and Technology, 7491 Trondheim, Norway; yaping.wang@ntnu.no

**Keywords:** nickel-based powder superalloy, constitutive model, hot compression

## Abstract

The deformation mechanism during the hot compression of PM nickel-based superalloy FGH99 and its micro-structural evolution, especially the evolution of γ’ phases, are the key factors affecting the final molding quality of aero-engine hot forged turbine disks. In this study, a new constitutive model of viscoplasticity with micro-structures as physical internal parameters were developed to simulate the hot compression behavior of FGH99 by incorporating the strengthening effect of the γ’ phase. The mechanical behavior of high-temperature (>1000 K) compressive deformation of typical superalloys under a wide strain rate (0.001~1 s^−1^) is investigated using the Gleeble thermal-force dynamic simulation tester. The micro-structure after the hot deformation was characterized using EBSD and TEM. Work hardening as well as dynamic softening were observed in the hot compression tests. Based on the mechanical responses and micro-structural features, the model considered the coupled effects of dislocation density, DRX, and γ’ phase during hot flow. The model is programmed into a user subroutine based on the Fortran language and called in the simulation of the DEFORM-3D V6.1 software, thus realizing the multiscale predictive simulation of FGH99 alloy by combining macroscopic deformation and micro-structural evolution. The established viscoplastic constitutive model shows a peak discrepancy of 10.05% between its predicted hot flow stresses and the experimental values. For the average grain size of FGH99, predictions exhibit an error below 7.20%. These results demonstrate the high accuracy of the viscoplastic constitutive model developed in this study.

## 1. Introduction

As a critical material for aero-engines, nickel-based superalloys play a very important role in aero-engine turbine disks and other critical hot end load-carrying components, owing to their superior high temperature performance [1,2]. Traditional cast superalloy ingots often exhibit coarse grains and severe dendritic segregation, making it difficult to achieve the fine-grained micro-structure required for high-performance components [3]. In contrast, powder metallurgy (PM) nickel-based superalloys offer inherent advantages in maintaining fine grain structures [4]. Through the hot isostatic pressing (HIP) technology to obtain high-density billet, billet isothermal forging is currently the mainstream PM nickel-based superalloy components preparation process. Nonetheless, the high deformation resistance coupled with a narrow processing window makes forging PM nickel-based superalloys significantly challenging during forging [5]. These superalloys are subjected to large loads, and the associated deformation heating can lead to localized temperature rises, resulting in micro-structural coarsening and potential defects [6]. The hot flow behavior of these alloys is highly complex and strongly influenced by temperature, strain, and strain rate [7]. Notably, plastic deformation’s role differs in transformations: Gurtin and Murdoch noted it can be neglected in solid–liquid surface stress tensor solving; for our study’s solid–solid deformation, it drives re-crystallization, altering the thermal field (1st Law) and forming new, less deformed surfaces. Therefore, accurately describing the thermal flow behavior and linking it to the evolution of micro-structures such as grains and dislocations is essential for the development, control, and optimization of the PM nickel-based superalloy forging processes.

Hot processing maps based on dynamic material models play an extremely critical role in optimizing forging process parameters by evaluating the superalloy response under different hot forming conditions. Tan et al. [8] established a hot processing map for Inconel 718 based on compression tests, identifying optimal parameters through research of power dissipation efficiency and flow instability. Similarly, Zhang et al. [9] constructed a map for ATI 718Plus using isothermal compression data and identified 1413~1453 K and 0.01~0.1 s^−1^ as the optimal range of temperature and strain rate for hot processing. However, although the workability of materials is visually enabled by processing maps, they only reflect behavior under specific conditions and lack the ability to capture the full coupling of flow behavior with micro-structural evolution during deformation.

Material constitutive modeling is an effective approach for investigating material behavior during hot deformation. It is generally classified into two types: phenomenological models based on empirical mathematical formulations and physically based constitutive models based on physical mechanisms. Phenomenological models focus on fitting experimental data to predict flow stress behavior within specific conditions. Kim [10] developed a predicted model to predict creep behavior of nickel-based superalloys based on mathematical laws; Chen [11] improved the Johnson–Cook model to describe hot flow behavior of FGH96. Meanwhile, Geng [12] constructed a dynamic re-crystallization (DRX) kinetics model using a modified Avrami equation. Although these models are efficient and accurate within limited conditions, they lack the ability to explain the physical mechanisms governing micro-structural evolution and flow behavior.

To overcome this limitation, physically based constitutive models have gained increasing attention. These models are built on fundamental principles such as thermodynamics and crystal plasticity and can reveal the intrinsic link between micro-structure and macroscopic flow behavior. Wu [13] derived a DRX prediction model for FGH96 with the Zener–Hollomon parameter as a functional expression based on thermal compression tests and quantitative micro-structure characterization. Slavik [14] incorporated internal variables like back stress and drag stress to capture rate sensitivity and creep behavior. Lin [15] proposed a two-stage physically based model for hot deformation. Basoalto [16] developed a multidimensional model considering damage and micro-structural evolution. The studies proved that these models could provide deep insights into the coupling mechanism between micro-structural evolution and macroscopic mechanical response of Inconel alloy 718 during hot deformation.

However, most existing physically based models mainly focus on a single micro-structural variable, such as DRX or damage, while ignoring critical factors like precipitates and grain size. In fact, precipitates have a significant effect on both hot deformation and micro-structural evolution. Zhang [17] reported that fine and nearly spherical precipitates pinned at grain boundaries reduce the DRX fraction around them, thereby influencing the overall macroscopic performance of GH4742.

In contrast to these approaches, the constitutive model proposed in this study accounts for the influence of multiple key microstructural variables such as re-crystallization, grain size, and dislocation density on macroscopic mechanical properties. It quantitatively accounts for the dissolution of γ’ precipitates with increasing temperature and their pinning effects on both grain boundaries and dislocation motion, thereby directly linking this essential microstructural feature to the macroscopic flow behavior. This multivariable physically coupled framework provides a more comprehensive and predictive description of the material’s thermo-mechanical response.

In this study, the hot flow behavior of PM nickel-based superalloy FGH99 was obtained based on high-temperature hot compression experiments; the main influence mechanisms within different strain stages are investigated, and the resulting micro-structure morphology of deformation-processed PM nickel-based superalloy FGH99, including grain size, dislocation density, and γ’ phase changes, are studied using electron backscattering diffraction (EBSD), transmission electron microscopy (TEM), and scanning electron microscopy (SEM). A new constitutive model of viscoplasticity with micro-structures as physical internal parameters was developed to predict the hot flow behavior of the FGH99 under different conditions by incorporating the strengthening effect of the γ’ phase. Secondary development of the constitutive model is realized through Fortran language to generate user subroutines applicable to DEFORM software and call them in DEFORM simulation, enabling high-accuracy simulation of both the micro-structural evolution and mechanical properties affected by precipitation phases and other microscopic influences during hot deformation of FGH99.

## 2. Experimental Methods

### 2.1. Experimental Material

**Figure 1:**
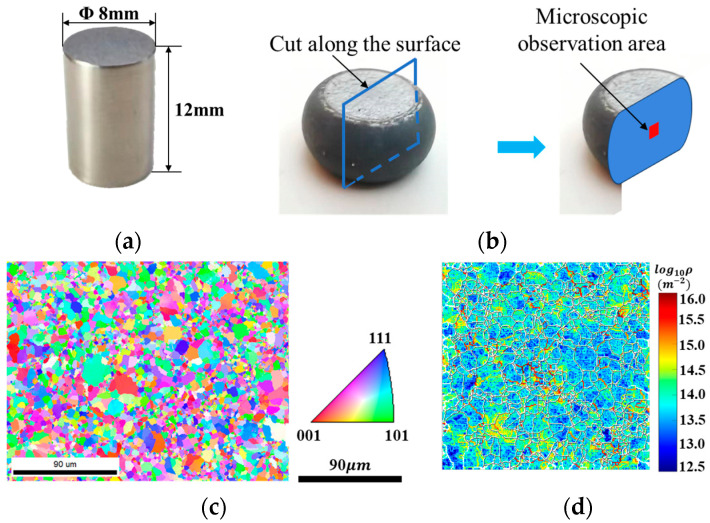
Specimens of hot compression tests. (**a**) Specimen dimensions; (**b**) micro-structure observation area; (**c**) EBSD observation of initial sample; (**d**) dislocation density of initial sample.

The standard cylindrical specimens used in hot compression tests are shown in Figure 1a, which were obtained by utilizing the wire-electrode cutting technique to cut the specimens from the disk billet along a direction parallel to the loading direction of the disk billet produced by hot isostatic pressing. These billets themselves are cylindrical blanks with a diameter of 300 mm and a height of 150 mm, prepared from the FGH99 alloy through vacuum induction melting, powder atomization, and hot isostatic pressing. The main chemical composition of FGH99 used in this experiment is listed in Table 1. Characterization of the initial micro-structure of the material using EBSD technology found that the grains of the initial specimen had an equiaxed grain structure, and the average grain size of FGH99 specimens calculated using area weighting was 6.3 μm (Figure 1c). The initial dislocation density of specimen was 7.8 × 10^13^ m^−2^, indicating a low level of prior plastic deformation.

### 2.2. Hot Compression Test

In this study, the Gleeble-3800 thermal simulator (Dynamic Systems Inc., Poughkeepsie, NY, USA) with induction heating was selected for the uniaxial isothermal hot compression tests to achieve uniform and stable heating of the specimen while reducing the impact on the specimen, and the corresponding conditions are given in Table 2. The temperature of the compressed specimen was accurately monitored and fed back to the testing system via a thermocouple, and the Gleeble closed-loop control system enabled rapid heating and maintained the specimen at a steady equilibrium temperature within ±1 K. The system also allowed the specimen to deform at a constant strain rate through precise control, ensuring the acquisition of accurate experimental data.

The thermocouple used for temperature measurement in this experiment is welded to the midpoint of the cylindrical specimen, enabling continuous monitoring throughout the experiment. A heating rate of 10 K/s was kept constant when heating the compression specimens to avoid temperature instability. Following attainment of set temperature, a 3 min soaking period was applied to specimens to further enhance temperature uniformity prior to deformation.

The parameters of the hot compression at different deformation levels are shown in Table 2 and Table 3. After the uniaxial compression test, to guarantee the precision of the characterized micro-structure, the superalloy compression specimen was cut along the central axis of the deformation direction, and a slice was taken in the center for micro-structure examination (Figure 1b). SEM was used to present the surface topographic features of the samples, while EBSD was used to obtain the crystallographic parameters of the samples and then characterize the micro-structural characteristics of the samples. MATLAB2019a was used to analyze the EBSD results and to plot the dislocation density maps of FGH99 under various hot compression conditions. TEM observation was conducted to investigate the γ’ phase after hot deformation.

## 3. Hot Compression Results

### 3.1. Hot Flow Behavior

**Figure 2:**
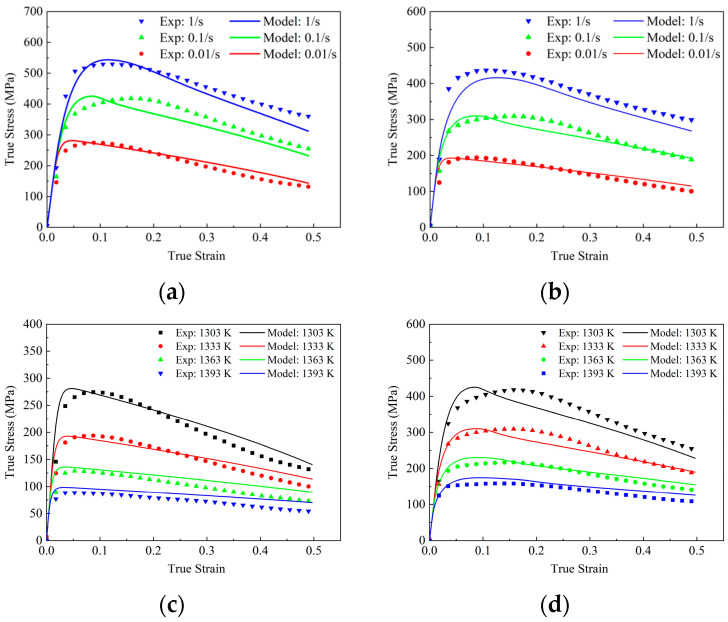
Stress–strain of hot compression tests at various temperatures and strain rates: (**a**) 1303 K; (**b**) 1363 K; (**c**) 0.01 s^−1^; (**d**) 0.1 s^−1^.

Figure 2 illustrates the stress–strain curve of FGH99 under hot compression conditions. The experimental data show that the dynamic recovery of FGH99 during hot compression gradually accelerates with the increase in the hot deformation temperature and the strain rate. This occurs because the elevated temperature reduces the driving force required for DRX and enhances dislocation generation and dislocation plugging by increasing strain rates, which makes the sample reach the critical dislocation density of DRX at lower strains, which leads to the early occurrence of DRX. By comparing the hot compression curves under various strain rates when the hot compression temperature is 1303 K and 1333 K, respectively (Figure 2a,b), it can be clearly seen that FGH99 is subjected to dynamic recovery during the compression process; when the stress reaches the peak, the curve will show a clear downward trend, which demonstrates the synergistic effect between hardening and softening during the compression process. This is caused by the micro-structure evolution during the material deformation process, where many more dislocations are generated during the deformation process, and dislocation proliferation and dislocation plugging impede the material deformation, with work hardening dominating in the early stage. With progressing specimen deformation, the DRX process accelerates. Concurrently, stress stabilizes when the rate of dislocation disappearance caused by dynamic restitution is almost equal to the rate of dislocation increase due to deformation. However, the stress of FGH99 does not remain stable but starts to decrease beyond the peak, attributed to the DRX occurs inside the alloy to consume the dislocations generated by deformation, thus leading to the decrease in stress [18].

Under a fixed strain rate, we investigated the effect of different compression temperatures on stress–strain behavior. A comparison of the rheological curves at different hot compression temperatures reveals that the maximum change in stress is nearly two times the value of the alloy at different compression temperatures, indicating that the alloy has a high sensitivity to temperature. As the compression proceeds, the curves at different compression temperatures show the same trend of change: the hot stress increases and then decreases as the compression temperature decreases. The peak stress increases from 215.15 MPa to 417.63 MPa as the hot compression temperature decreases from 1393 K to 1303 K. This is due to the fact that the higher temperature increases the dislocation climbing and slipping ability, so the peak stress is relatively small.

### 3.2. Micro-Structure Characterization

**Figure 3:**
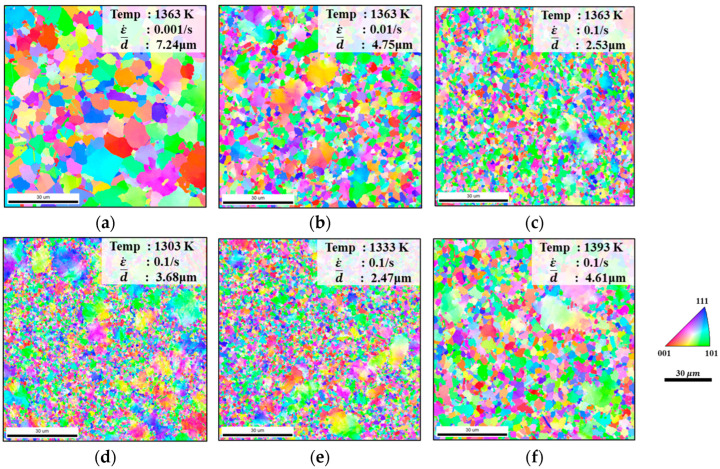
EBSD micro-structure observations at 1363 K; engineering strain is 0.5 under (**a**) $\dot{\varepsilon }$ = 0.001 s^−1^, (**b**) $\dot{\varepsilon }$ = 0.01 s^−1^, and (**c**) $\dot{\varepsilon }$ = 0.1 s^−1^, and when $\dot{\varepsilon }$ = 0.1 s^−1^, engineering strain is 0.5 with various temperatures (**d**) 1303 K, (**e**) 1333 K, and (**f**) 1393 K.

**Figure 4:**
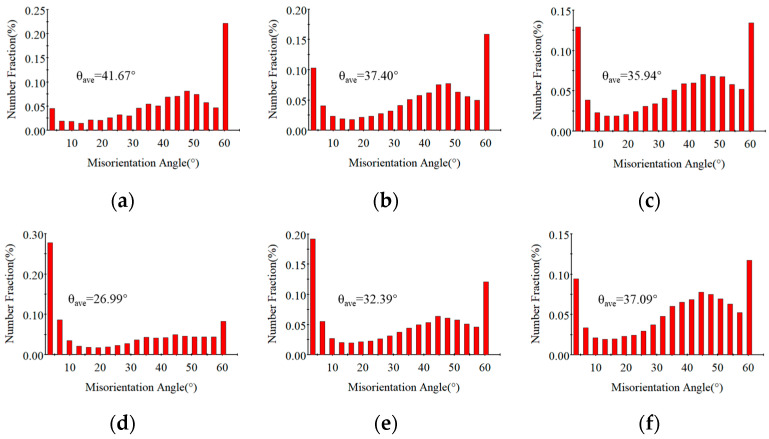
Statistical map of grain boundary orientation difference at 1363 K; engineering strain is 0.5 under (**a**) $\dot{\varepsilon }$ = 0.001 s^−1^, (**b**) $\dot{\varepsilon }$ = 0.01 s^−1^, and (**c**) $\dot{\varepsilon }$ = 0.1 s^−1^. and when $\dot{\varepsilon }$ = 0.1 s^−1^, engineering strain is 0.5 with various temperatures (**d**) 1303 K, (**e**) 1333 K, and (**f**) 1393 K.

The grain size and DRX evolution pattern are the key to understanding how micro-structure influences the hot flow of FGH99. In this study, the micro-structure of hot compression samples under different conditions was characterized using EBSD (Figure 3). Figure 3a–c shows the micro-structure grain morphology of FGH99 at 1363 K. Under this condition, elevated strain rates result in finer DRX grains Higher strain rates drive this refinement by intensifying dislocation multiplication and the formation of dislocation cells. The rapid proliferation of dislocations, particularly of edge dislocations that can only move in one direction, tends to concentrate at grain boundaries or dislocation pile walls, forming stable tangles and dislocation forests that provide a high density of potential nucleation sites for DRX [19]. The high energy of these dislocation cell boundaries facilitates nucleation, thereby increasing the nucleation rate and resulting in finer grains after DRX. Additionally, the reduced deformation time inherent to higher strain rates restricts the thermally activated recovery processes. Specifically, the cross-slip of screw dislocations and the climb of edge dislocations are suppressed, resulting in insufficient subgrain growth. Consequently, low-angle grain boundaries have limited mobility and cannot effectively transform into high-angle grain boundaries (HAGBs), thereby suppressing the growth of re-crystallized grains. Figure 4a–c also illustrates that the proportion of HAGBs decreases as the strain rate increases. Sustained DRX during processing leads to the presence of fine DRX grains in the material at the end of the test along with the grown re-crystallized coarse crystals. This is because the dissolution of the γ’ phase under high-temperature conditions weakens Zener pinning, allowing grains with lower interfacial energy to consume fine particles. Additionally, due to the low local strain, re-crystallization is difficult to occur, and grain growth continues.

We compared the micro-structures under different temperatures ranging from 1303 K to 1393 K under a fixed strain rate (Figure 3d–f). When the compression temperature is 1303 K (Figure 3d), re-crystallization was incomplete due to insufficient driving force, resulting in the retention of a large number of primary grains. Furthermore, strain-induced grain growth triggered by uneven deformation distribution further increased the variation in grain size. The grain size at 1303 K is larger than that at 1333 K. This is because, on the one hand, the decrease in temperature reduces the driving force for re-crystallization, allowing a higher proportion of the original coarse grains to remain in the micro-structure; on the other hand, as the temperature decreases, the number of re-crystallization nucleation sites increases considerably to minimize the Gibbs-Free Energy, and as under-cooling increases, the growth is restrained by lower diffusion, thereby leading to the formation of numerous fine grains in the material. Meanwhile, the suppressed mobility of both screw and edge dislocations at lower temperatures restrains boundary migration, leading to the formation of numerous fine grains that only account for a small proportion, such that the overall average grain size remains large. When the temperature reaches 1333 K, the grain size exhibits a gradual increase with rising temperature. It can be deduced that within the temperature range of 1303~1333 K at 0.1 s^−1^, specific temperature points exert a pronounced influence on the occurrence of re-crystallization, with the re-crystallization rate increasing significantly under these thermal conditions. This increase is accompanied by the gradual elimination of dislocations, leading to a progressive weakening of work hardening. As the temperature is gradually raised to 1393 K, the high-temperature effect promotes grain boundary migration while simultaneously causing the extinction of most dislocations. This results in a significant reduction in flow stress, indicating that both grain growth and dislocation evolution exhibit high thermal sensitivity.

**Figure 5:**
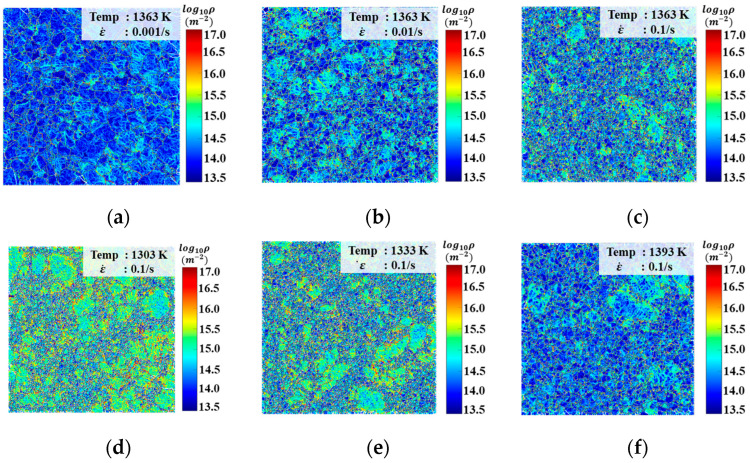
GND density comparison at 1363 K; engineering strain is 0.5 under (**a**) $\dot{\varepsilon }$ = 0.001 s^−1^, (**b**) $\dot{\varepsilon }$ = 0.01 s^−1^, and (**c**) $\dot{\varepsilon }$ = 0.1 s^−1^, and when $\dot{\varepsilon }$ = 0.1 s^−1^, engineering strain is 0.5 with various temperatures (**d**) 1303 K, (**e**) 1333 K, and (**f**) 1393 K.

To analyze the dislocation evolution behavior of FGH99 during hot compression, EBSD data were processed using MATLAB to obtain geometrically necessary dislocation (GND) density under different compression conditions. The dislocation density distributions are presented in Figure 5 for samples with an engineering strain of 0.5 at various conditions. At a compression temperature of 1363 K (Figure 5a–c), a rise in strain rate corresponds to a substantial increase in the dislocation density. Elevated strain rates shorten the time window for DRX to occur, leading to insufficient dislocation annihilation and consequently causing dislocation accumulation within the material. As a result, the overall dislocation density increases. Additionally, Figure 5a clearly shows that dislocations tend to accumulate at the boundaries of coarse grains, which is attributed to the consumption of stored dislocations near coarse grain boundaries during re-crystallization. Comparing the GND density distributions under different deformation temperatures (Figure 5c–f) at 0.1 s^−1^, obviously GND density decreases significantly with increasing temperature. This is because elevated temperatures enhance dislocation climb, promote the onset of DRX, and accelerate the rearrangement and annihilation of dislocations.

**Figure 6:**
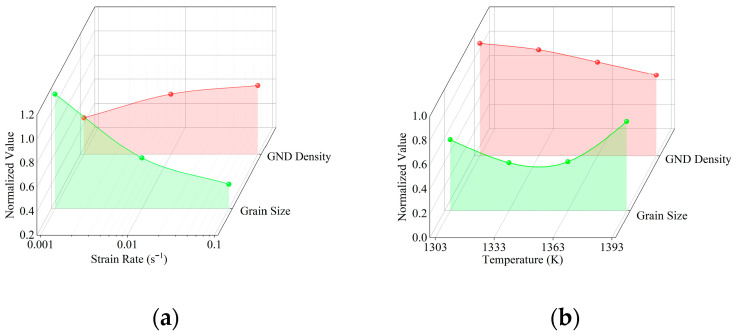
Effect under (**a**) temperature at 1363 K, (**b**) $\dot{\varepsilon }$ = 0.1 s^−1^ on grain size and GND density.

Comparison of dislocation density and grain size under the same conditions reveals a strong negative correlation between grain size and GND density under constant deformation temperature or strain rate (Figure 6). Elevated strain rates limit the time available for DRX to consume dislocations, causing dislocations to accumulate rapidly. Therefore, when the compression temperature is 1363 K (Figure 6a), increasing the strain rate not only elevates GND density but also promotes grain refinement. The regions with high dislocation density store higher energy, thereby activating DRX nucleation, ultimately leading to grain refinement as the strain rate increases. At a fixed strain rate, as the temperature increases, the density of GND decreases gradually, while the normalized grain size of FGH99 decreases and then increases, reaching the lowest value at 1333 K (Figure 6b). This occurs because higher temperatures promote both the nucleation of new grains and their subsequent growth. Otherwise, elevated temperatures can dissolve precipitates, relieving dislocation pile-up. Meanwhile, newly formed re-crystallized grains possess lower dislocation densities, resulting in an overall reduction in GND density.

**Figure 7:**
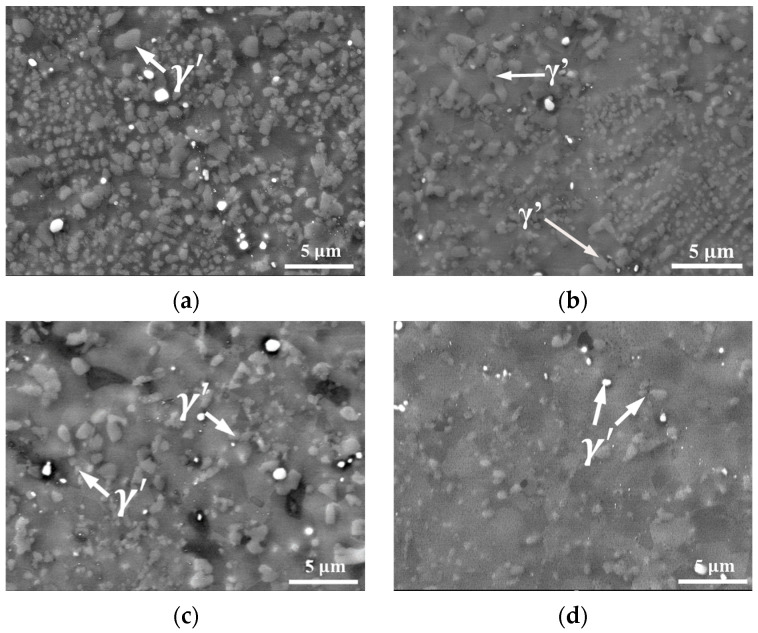
SEM micrographs of FGH99 at various temperatures of (**a**) 1303 K; (**b**) 1333 K; (**c**) 1363 K; (**d**) 1393 K.

**Figure 8:**
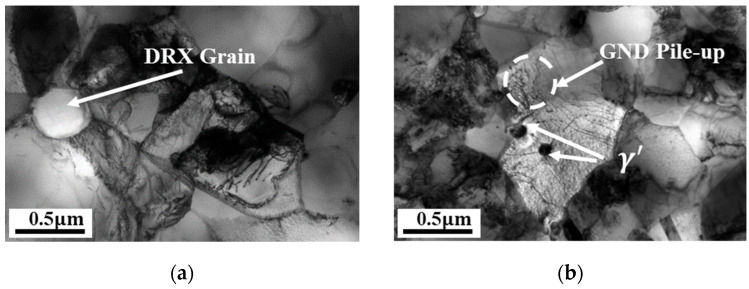
TEM images of FGH99 at strain rates of (**a**) 0.01 s^−1^; (**b**) 0.1 s^−1^ under 1363 K.

As observed in the SEM micrographs in Figure 7, the content of the γ’ phase significantly decreases with the temperature increasing from 1303 K (Figure 7a) to 1393 K (Figure 7d). Using Image-Pro Plus 6.0 software to measure the volume fraction of the precipitated phase at different temperatures and strain rates of 0.1/s revealed that the γ’ volume fraction drops sharply from approximately 62.9% at 1303 K to 22.2% at 1333 K (Figure 7b). This substantial dissolution directly weakens the pinning effect of the precipitates on the grain boundaries. As a result, grain growth is promoted and dislocation density decreases with increasing temperature, as previously discussed. The pinning effect continues to diminish as the γ’ volume fraction further decreases to 15.2% at 1363 K and 5.4% at 1393 K. At the same time, considering the interaction between the γ’ phase and dislocations, the effect on γ’ distribution in response to steady-state dislocation nucleation should not be neglected during γ’ nucleation and is taken into account in the subsequent modeling. Figure 8 shows TEM images at a compression temperature of 1363 K. The newly re-crystallized grains exhibit very low internal GND density, with most dislocations concentrated at the grain boundaries of the new grains (Figure 8a). Figure 8b reveals γ’ precipitates located at and within the grain boundaries, with dislocations distributed around them. The pinning effect of the γ’ phase on the grain boundaries results in a serrated boundary morphology, which enhances the resistance of grain boundaries to deformation at high temperatures. Additionally, dislocation pile-up caused by the impediment of γ’ to dislocation motion can also be observed in the figure.

## 4. Material Modeling

### 4.1. Constitutive Modeling of Nickel-Based Powder Superalloy

In this study, we developed a constitutive model of viscoplasticity with micro-structures as physical internal parameters to simulate the hot compression behavior of FGH99 by incorporating the strengthening effect of the γ’ phase and dislocation density. The model describes the hot flow behavior and the associated micro-structural evolution, including grain size, re-crystallization, and dislocation density. The equations are formulated based on the Euler integration method to update internal state variables, and the state variable evolution is represented through a set of rates equations [20].

#### 4.1.1. Modeling of γ’ Precipitates

The particle size and volume fraction size within FGH99 for γ’ exerts a significant strengthening effect on nickel-based superalloys. Given the low strain rates utilized in our experiments and our primary focus on phase nucleation and growth processes post-hot compression instead of dynamic nucleation occurring during deformation, Classical Nucleation Theory (CNT) was employed with appropriate simplifications to analyze the γ’ phase nucleation behavior. Equation (1) describes the time-dependent growth behavior of precipitates with different dimensions based on Zener theory [21]:

(1) $$r=\alpha_{\lambda }{\left(Dt\right)}^{1/2}$$

where D denotes the solute diffusion rate (primarily Al and Ti diffusion in the γ matrix) with temperature dependence; it describes concentration gradient-driven solute migration and follows the Arrhenius equation. $\alpha_{\lambda }$ is the precipitated phase growth system, t is the time. During the precipitation process, $\alpha_{\lambda }$ is affected by the concentration of solute atoms, and according to the mass conservation of precipitated elements, it can be considered that the growth coefficient is related to the volume fraction of the precipitated phase [22]. From the mathematical relationship, the precipitated phase size r is inversely proportional to time t, and it can be considered that r should be exponentially related to time t according to Equation (1).

The increase in dislocation density induced by deformation can significantly influence the growth behavior of the precipitated phase [23]. Meanwhile, once the precipitate size reaches a critical value, further growth is impeded due to capillary effects [24]. Based on this consideration, the growth rate of the γ’ phase, $\dot{r}$ can be expressed as follows:

(2) $$\dot{r}=\frac{k_{3}}{2}{\left(\frac{D}{r^{d}}f_{r}\right)}^{\frac{1}{2}}(1-\frac{r}{r_{c}})(1+k_{2}{\bar{\rho }}^{n_{3}}\dot{\bar{\rho }})$$

where $k_{3}$ is a constant related to solute concentration, d is the exponential constant of the γ’ size, $r_{c}$ is the critical radius of γ’ particles, and $k_{2},n_{3}$ are material constants. $\bar{\rho }$ and $\dot{\bar{\rho }}$ denote the average dislocation density and its rate of change, respectively.

According to classical nucleation theory, the precipitation process of a secondary phase from a supersaturated matrix is governed by nucleation kinetics [25], and the growth is further affected by the evolving volume fraction of the precipitates [26]. Equation (3) describes the evolution of the γ’ phase volume fraction, which is influenced by both γ’ phase volume and nucleation rate [27].

(3) $${\dot{f}}_{r}=S_{1}r^{3}\frac{R_{1}Z(1-p){\bar{\rho }}^{m_{0}}{\left(1-\frac{f_{r}}{f_{r}^{*}}\right)}^{m_{1}}{\varepsilon^{p}}^{m_{2}}}{b}$$

where $S_{1}$ is the volume scaling factor of γ’, $R_{1}$ is a material constant, *p* is the non-nucleation probability of precipitates, *Z* is the Zeldovich factor, and $m_{0}$, $m_{1}$, $m_{2}$ are material constants. $\varepsilon^{p}$ is the equivalent plastic strain, and $f_{r}^{*}$ denotes the saturation volume fraction of γ’.

The strengthening effect of γ’ on the material can be described by following relation [28]:

(4) $$\sigma_{p}=C_{3}{\left(q_{5}f_{r}\right)}^{n_{4}}r^{n_{5}}$$

where $C_{3}$ and $q_{5}$ are the material constants. The precipitation strengthening contribution $\sigma_{p}$ directly influences the initial yield stress.

#### 4.1.2. Constitutive Modeling of Hot Deformation

Equation (5) represents the strain rate relationship, where the stress–strain behavior follows a power-law form. This equation is the criterion for the viscoplasticity of the material expressed by the strain rate equation [29]:

(5) $${\dot{\varepsilon }}^{p}={\left[\left(\sigma -R-k\right)/K\right]}^{n_{1}}{\left(\bar{d}\right)}^{-\mu }$$

where ${\dot{\varepsilon }}^{p}$ denotes the plastic strain rate, *R* is the relevant parameter for measuring isotropic hardening, σ is the equivalent stress, *k* is the initial yield stress expressed as $k=\sigma_{0}+\sigma_{p}$, *K* is the strength coefficient, $\bar{d}$ is average grain size, and $n_{1}$ is the strain hardening exponent; μ is the material constant.

Equation (6) defines the dislocation density $\bar{\rho }$. Material dislocation accumulation is mainly related to plastic deformation. As deformation proceeds, both dynamic recovery and DRX consume dislocations, and the dislocation density gradually stabilizes during this process [30]:

(6) $$\dot{\bar{\rho }}=A\left(1-\bar{\rho }\right)\left|{\dot{\varepsilon }}^{p}\right|-C_{1}{\bar{\rho }}^{n2}-\left[\left.C_{2}\bar{\rho }/(1-S\right)\right]\dot{S}$$

where $\dot{S}$ is the re-crystallization growth rate; $C_{1}$, $C_{2}$ and $n_{2}$ are material constants.

Equation (7) is the critical dislocation density equation [31]:

(7) $${\bar{\rho }}_{c}=q_{3}{\dot{\varepsilon }}^{q_{4}}$$

Equation (8) is the hardening rate equation [32]:

(8) $$\dot{R}=0.5B{\bar{\rho }}^{-0.5}\dot{\bar{\rho }}$$

Equation (9) is the evolution equation of average grain size [33]:

(9) $$\dot{d}=w_{1}d^{-\gamma_{1}}+w_{2}{\dot{\varepsilon }}^{-\gamma_{2}}-w_{3}{\dot{S}}^{\gamma_{3}}{\left(\bar{d}\right)}^{\gamma_{4}}$$

where $w_{1}$, $w_{2}$, $w_{3}$, $\gamma_{1}$, and $\gamma_{2}$ are material constants.

Equation (10) represents the evolution of DRX. When dislocations reach critical dislocations, the DRX gestation period varies with the dislocation density [33]. The DRX volume fraction S varies in the range of 0 to 1:

(10) $$\dot{S}=Q_{0}\left[x\bar{\rho }-{\bar{\rho }}_{c}\left(1-S\right)\right]{\left(1-S\right)}^{N_{q}}$$

where *x* is the incubation time for re-crystallization.

Equation (11) is the equation for the re-crystallization inoculation time [33]:

(11) $$\dot{x}=A(1-x)\bar{\rho }$$

Equation (12) is the uniaxial damage rate equation [34]:

(12) $$\dot{\omega }=\frac{\eta_{1}{\dot{\varepsilon }}^{\eta_{2}}}{{\left(1-\omega \right)}^{\eta_{3}}}$$

where $\eta_{2}$, $\eta_{3}$ are material constants.

Equation (13) is the material elastic–plastic stress–strain equation [34]:

(13) $$\sigma =E(1-\omega )(\varepsilon^{T}-\varepsilon^{P})$$

where E is Young’s modulus; $\varepsilon^{T}$ is the total strain.

In the equations, $n_{4}$, $n_{5}$, *k*, *K*, *A*, $q_{3}$, $q_{4}$, B, $\gamma_{3}$, $\gamma_{4}$, $Q_{0}$, $N_{q}$, $A_{1}$, and $\eta_{1}$ are temperature-dependent parameters. Table 4 shows the temperature-dependent parameters, which were expressed as the Arrhenius equation [35].

### 4.2. Model Verification

#### 4.2.1. Prediction of Stress–Strain Curve

Figure 2 compares the predicted stress–strain curve obtained from the constitutive model (solid curves) with the experimental data (symbols). The experimental results were obtained based on hot compression experiments and were represented as scatter points, and the model prediction curves were obtained by iterative solving with the Runge–Kutta method and are represented as solid lines in the image. It is evident that the predicted stress–strain curves align with the actual curves in terms of trend, with distinct work hardening and softening phenomena observable. These characteristics are primarily achieved through the adjustment of the model’s dislocation and DRX equations.

To quantitatively evaluate the predictive accuracy of the model, the Pearson correlation coefficient R was calculated using Equation (14):

(14) $$R=\frac{\sum_{i=1}^{N}\left(\sigma_{E}-{\bar{\sigma }}_{E})(\sigma_{P}-{\bar{\sigma }}_{P}\right)}{\sqrt{\sum_{i=1}^{N}{\left(\sigma_{E}-{\bar{\sigma }}_{E}\right)}^{2}\sum_{i=1}^{N}{\left(\sigma_{P}-{\bar{\sigma }}_{P}\right)}^{2}}}$$

where $\sigma_{E}$ and $\sigma_{P}$ are the experimental and predicted stress values, ${\bar{\sigma }}_{E}$ and ${\bar{\sigma }}_{P}$ are their corresponding mean value.

The calculated R values under different temperature conditions are 0.9589, 0.9639, 0.9572, and 0.9384, respectively, with an average prediction accuracy of 0.9479; at a temperature of 1303 K and a strain rate of 0.1 s^−1^, the model exhibits a minimum predicted accuracy of 89.95%, indicating that its predictive accuracy exceeds 89.95%, demonstrating that the established constitutive models possess good predictive capability for flow stress. The equations of temperature-dependent parameters are summarized in Table 4. The determined constants are listed in Table 5.

#### 4.2.2. Prediction of Grain Size

**Figure 9:**
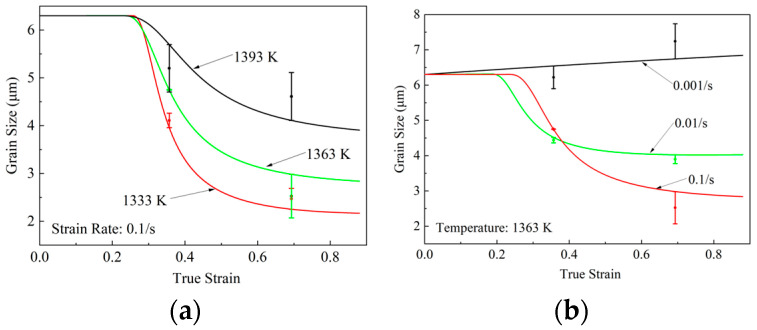
Comparison of experimental and predicted grain size (**a**) with various temperatures at 0.1 s^−1^; (**b**) with various strain rates at 1363 K.

Figure 9 presents the model-predicted average grain size of FGH99 across compression conditions. Figure 9a presents the predicted grain size at temperatures of 1333 K, 1363 K, and 1393 K at 0.1 s^−1^, while Figure 9b shows the grain size predictions at 1363 K over a range of strain rates. Figure 9a demonstrates that grain size expands at higher temperatures, reflecting the role of temperature in promoting static grain growth as modeled in Figure 9b. At lower strain rates, longer deformation times allow more time for grain growth following re-crystallization, resulting in larger average grain sizes. These predictions are consistent with experimental observations. The average predicted deviations of grain sizes are 8.53% and 5.87%, respectively. All predicted values fall within the experimental error bars, further confirming the accuracy of the model.

## 5. Deform Thermal Compression Simulation

### 5.1. Buildup of Model

**Figure 10:**
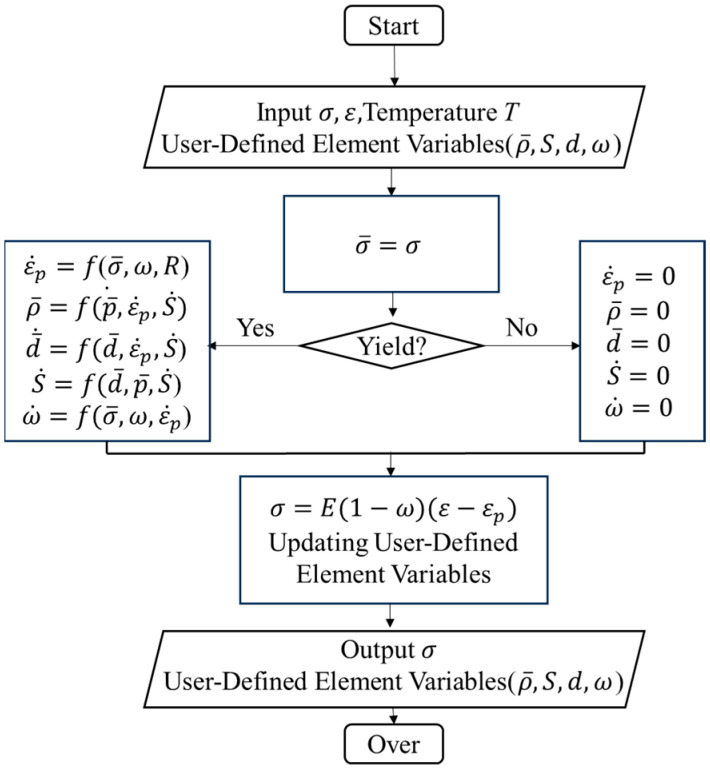
Flowchart of secondary development of constitutive models.

A FE model of the hot compression process was developed using DEFORM-3D V6.1-software. The physically based constitutive model of FGH99 was embedded through user-defined subroutines. Before embedding the model as a user subroutine in DEFORM software, relevant user-defined element variables must be defined for the viscoplastic constitutive model. This facilitates observing the distribution of corresponding macroscopic mechanical properties and microstructural variables in the post-processing module. Table 6 lists the meanings of user-defined variables. The flowchart for incorporating the viscoplastic constitutive model into a subroutine is shown in Figure 10.

**Figure 11:**
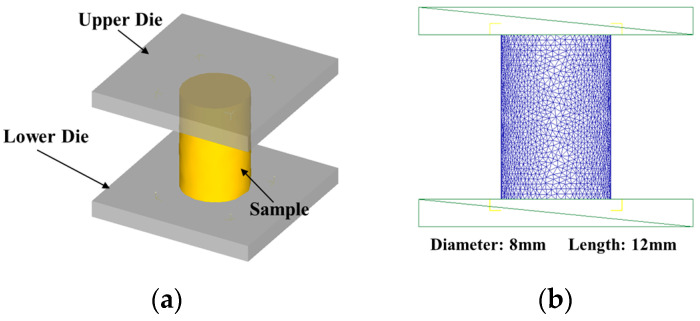
FE model of the hot compression process: (**a**) 3D model; (**b**) meshing configuration of the specimen.

Figure 11a shows the 3D model of the specimen positioned within the upper and lower mold configuration. The specimen dimensions match those used in the hot compression test. A global Cartesian coordinate system was defined, with the compression axis aligned along the Z-direction. After importing the STL-format geometric model into the Deform preprocessing section, the following process and run parameters were set: First, the mold is defined as a rigid body without meshing, and the workpiece is defined as a plastic body. Automatic meshing is then performed, with the mesh count set to 16,000. Next, secondary development is performed on the constitutive model derived in Section 4.1 to establish a material library for FGH99, which is then assigned to the workpiece. Simultaneously, based on hot stamping principles, a shear friction of 0.1 is applied for the interaction between the workpiece and mold. Since the simulation involves isothermal forging, the thermal conductivity coefficient is set to 0. Contact is established by placing the upper and lower molds in contact with the workpiece, thereby completing the contact configuration. Additionally, for model constraints, the lower die was fixed, while the upper die’s pressing speed was set to follow the displacement defined by Equation (15). This ensured a constant strain rate consistent with experimental conditions. The initial temperatures of the specimen and dies were set based on experimental data. The minimum time step is set to 0.01 times the unit’s downward displacement height. The number of simulation steps is defined as the ratio of the downward displacement height to the minimum time step. Data is saved every 50 steps, a DB file is generated, and the simulation model is run.

(15) $$v=h_{0}\dot{\varepsilon }e^{-\dot{\varepsilon }t}$$

where *v* is the upper die velocity, *t* denotes the compression time in the simulation, $h_{0}$ is the initial specimen height, and $\dot{\varepsilon }$ is the strain rate.

The distribution of grain size and re-crystallized fraction within the specimens was then obtained from the simulation.

### 5.2. Finite Element Results and Analysis

**Figure 12:**
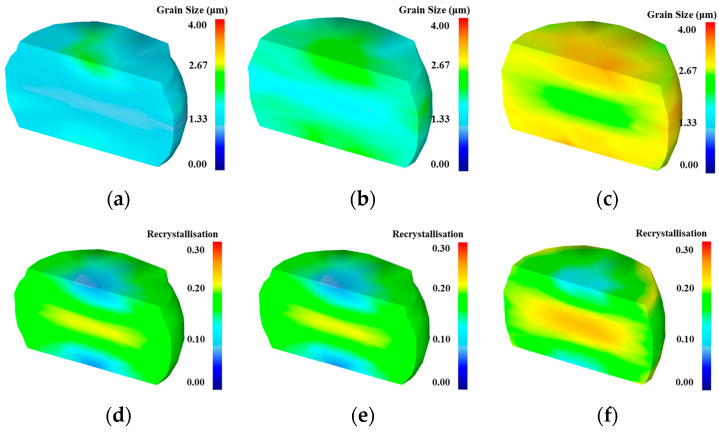
Simulated results when $\dot{\varepsilon }$ = 0.1 s^−1^: grain size at (**a**) 1333 K; (**b**) 1363 K; (**c**) 1393 K; DRX fraction at (**d**) 1333 K; (**e**) 1363 K; (**f**) 1393 K.

As shown in Figure 12, the grain size contour map and re-crystallization contour map simulated by DEFORM-3D V6.1 software under different temperature conditions with a strain rate of 0.1/s are presented. From the perspective of individual specimens, billets exhibit a barrel shape after upsetting, with a high degree of re-crystallization in the central region, small average grain size, and significantly refined microstructure. In areas contacting the die, friction results in minimal deformation, a low re-crystallization fraction, and insignificant grain refinement. From the perspective of temperature, with the increase in temperature, the overall grain size of the sample gradually increases. Meanwhile, under different conditions, the grain size in the central area is smaller than that in the surrounding areas. This is mainly because during the hot compression process, the isotropic effect changes significantly in the central region, and re-crystallization is more complete compared to the periphery, resulting in a smaller grain size. With the increase in temperature, the re-crystallization fraction generally shows an upward trend. This is because the increase in temperature promotes the diffusion of atoms, and at the same time, high temperature provides more activation energy to promote the occurrence of material re-crystallization. However, as the re-crystallization fraction increases, the grain size decreases because the high temperature promotes the growth of grains, resulting in the phenomenon that the higher the temperature, the larger the re-crystallization fraction and the larger the grain size. These simulation results align with micro-structural evolution observed in hot compression experiments conducted in this study.

## 6. Conclusions

In this paper, a constitutive model of viscoplasticity with micro-structures as physical internal parameters for FGH99 was developed, with consideration of the influence of γ’ on the hot compression behavior of the FGH99. The study leads to the following principal conclusions:

(1) Within the temperature range of 1303–1393 K, FGH99 exhibit high sensitivity to both strain rate and temperature. As temperature increases and strain rate decreases, the flow stress rises. Significant work hardening occurs during the early stages of deformation, attributed to dislocation multiplication and dislocation pile-up hindering the deformation of the alloy. As deformation progresses, dynamic recovery becomes one of the dominant mechanisms governing the material’s behavior.
(2) Microstructural observations by EBSD indicate that the hot deformation behavior of FGH99 is governed by the competitive interaction between work hardening and DRX. Elevated strain rates promote dislocation accumulation and grain refinement by restricting recovery and DRX progress, whereas higher temperatures facilitate dislocation annihilation and grain growth. There is a strong negative correlation between grain size and GND density.
(3) TEM observations reveal the γ’ phase as a strengthening phase in FGH99. Microscopic morphology indicates that γ’ significantly impedes dislocation movement. Concurrently, the precipitation of γ’ along grain boundaries results in a serrated surface structure at these interfaces. This enhances the creep resistance of nickel-based superalloys during high-temperature deformation. Furthermore, the inherently high shear strength of the γ’ phase exerts a pronounced influence on the mechanical properties of the material.
(4) A new constitutive of viscoplasticity with micro-structures as physical internal parameters was established, which is able to accurately predict the stress–strain curve of FGH99 in the experimental range, with a maximum deviation of 10.05% in its prediction. The grain size was also predicted with a maximum deviation of 7.20%. In addition, through the secondary development to achieve the call of the model in the DEFORM simulation, the micro-structure evolution in the simulation is consistent with the micro-structure pattern in the test.

## Figures and Tables

**Table 1:** Chemical composition of nickel-based powder superalloys (wt.%).

Cr	Co	Mo	Ta	Nb	Al	Ti	C	Zr	B	Ni
11.0~13.0	19.0~22.0	3.5~6.0	2.4~4.0	0.5~1.0	3.0~5.0	0.0~4.5	0.05	0.05	0.03	44.1~60.47

**Table 2:** Experimental parameters of hot compression at different strain rates and temperatures when engineering strain is 0.3 (the √ in the list indicate completed experiments).

	0.001 s^−1^	0.01 s^−1^	0.1 s^−1^	1 s^−1^
1303 K	√	√	√	√
1333 K	√	√	√	√
1363 K	√	√	√	√
1393 K	√	√	√	√

**Table 3:** Experimental parameters of hot compression at different strain rates and temperatures when engineering strain is 0.5 (the √ in the list indicate completed experiments).

	0.001 s^−1^	0.01 s^−1^	0.1 s^−1^	1 s^−1^
1303 K	√	√	√	√
1333 K	√	√	√	√
1363 K	√	√	√	√
1393 K	√	√	√	√

**Table 4:** Equations of temperature-dependent parameters.

$k=k_{0}exp(\frac{Qk_{0}}{RT})$	$K=K_{0}exp(\frac{QK_{0}}{RT})$	$q_{3}=q_{30}exp(\frac{Qq_{30}}{RT})$	$q_{4}=q_{40}exp(-\frac{Qq_{40}}{RT})$
$Q_{0}=Q_{00}exp(\frac{QQ_{00}}{RT})$	$N_{q}=N_{q0}exp(\frac{QN_{q0}}{RT})$	$A=A_{0}exp(\frac{QA_{0}}{RT})$	$\gamma_{3}=\gamma_{30}exp(-\frac{Q\gamma_{30}}{RT})$
$\gamma_{4}=\gamma_{40}exp(-\frac{Q\gamma_{40}}{RT})$	$B=B_{0}exp(\frac{QB_{0}}{RT})$	$\eta_{1}=\eta_{10}exp(\frac{Q\eta_{10}}{RT})$	$A_{1}=A_{10}exp(-\frac{QA_{10}}{RT})$

**Table 5:** Determined constants for the constitutive equations for FGH99.

$K_{0}$ (MPa)	$k_{0}$ (MPa)	$q_{3}$	$q_{4}$	$Q_{00}$	$N_{q0}$
0.14667	1.39 × 10^−8^	0.00478	47.7141	2.96 × 10^−5^	7.267 × 10^−6^
$A_{0}$	$\gamma_{3}$	$\gamma_{4}$	$B_{0}$ (MPa)	$\eta_{10}$	$A_{10}$
2.863 × 10^−6^	4.490 × 10^4^	42.044	0.0532	5.594 × 10^−4^	2.484 × 10^4^
$QK_{0}$ (J/mol)	$Qk_{0}$ (J/mol)	${Qq}_{30}$ (J/mol)	${Qq}_{40}\;(J/mol)$	${QQ}_{00}\;(J/mol)$	$QN_{q0}\;(J/mol)$
7.28 × 10^4^	2.480 × 10^5^	5.156 × 10^4^	5.162 × 10^4^	1.210 × 10^5^	1.599 × 10^5^
$QA_{0}$ (J/mol)	$Q\gamma_{30}\;(J/mol)$	$Q\gamma_{40}\;(J/mol)$	$QB_{0}$ (J/mol)	$Q\eta_{10}\;(J/mol)$	$QA_{10}$ (J/mol)
1.768 × 10^5^	1.161 × 10^5^	2.981 × 10^4^	9.5671 × 10^4^	7.870 × 10^4^	1.202 × 10^5^
$n_{1}$	μ	$C_{1}$ ($s^{-1}$)	$n_{2}$	$C_{2}$ ($s^{-1}$)	$w_{1}$
3.5649	2.1	3.30675	3.5649	0.1016	0.02
$w_{2}$	$w_{3}$	$\gamma_{1}$	$\gamma_{2}$	$\eta_{10}$	$\eta_{2}$
3.4 × 10^−3^	1.39451	3.92391	1.8529	5.594 × 10^−4^	0.98
$\eta_{3}$	*E* (MPa)	$n_{3}$	$R_{1}$	b	$n_{5}$
0.7	1.337 × 10^4^	0.21	1 × 10^−5^	2 × 10^3^	2.2
Z	$n_{4}$	$m_{0}$	$m_{1}$	$m_{2}$	P
2.3	8	0.02	2.2	0.01	0.3
$k_{2}$	$k_{3}$	*D*	*c*	$q_{5}$	$C_{3}$
4	12	1 × 10^3^	26	1.2	2.5 × 10^4^

**Table 6:** The user-defined element variables.

User-Defined Element Variables	Symbol	Variable Definition
USRE1 (1)	$\varepsilon^{P}$	Equivalent Plastic Strain
USRE1 (2)	${\dot{\varepsilon }}^{p}$	Equivalent Plastic Strain rate
USRE1 (3)	R	Work Hardening
USRE1 (4)	$\bar{\rho }$	Normalized Dislocation Density
USRE1 (5)	$\bar{d}$	Grain Size
USRE1 (6)	S	Re-crystallization Fraction
USRE1 (7)	${\bar{\rho }}_{c}$	Normalized Critical Dislocation Density
USRE1 (8)	ω	Damage Coefficient
USRE1 (9)	$\dot{\bar{\rho }}$	Dislocation Density Rate
USRE1 (10)	$\dot{R}$	Work Hardening Rate
USRE1 (11)	$\dot{S}$	Re-crystallization Rate
USRE1 (12)	x	Re-crystallization inoculation time

## Data Availability

The original contributions presented in this study are included in the article. Further inquiries can be directed to the corresponding author.
